# Mini-P-gp and P-gp Co-Expression in Brown Trout Erythrocytes: A Prospective Blood Biomarker of Aquatic Pollution

**DOI:** 10.3390/diagnostics5010010

**Published:** 2015-01-12

**Authors:** Emeline Valton, Christian Amblard, François Desmolles, Bruno Combourieu, Frédérique Penault-Llorca, Mahchid Bamdad

**Affiliations:** 1 ERTICa—EA 4677, Département Génie Biologique, Institut Universitaire de Technologie, Clermont Université—Université d’Auvergne, Ensemble universitaire des Cézeaux, B.P. 86-63172 Aubiere, France; E-Mail: emeline.valton@gmail.com; 2 ERTICa—EA 4677, Centre Jean Perrin, Clermont Université—Université d’Auvergne, 58 rue Montalembert, B.P. 392-63011 Clermont-Ferrand, France; E-Mail: frederique.penault-llorca@cjp.fr; 3 LMGE—UMR CNRS 6023, Clermont Université—Université Blaise Pascal, B.P. 80026-63171 Aubiere, France; E-Mail: christian.amblard@univ-bpclermont.fr; 4 Fédération pour la Pêche et la Protection du Milieu Aquatique du Puy de Dôme (F.P.P.M.A. 63), 14 Allée des Eaux et Forêts, Site de Marmilhat Sud, 63370 Lempdes, France; E-Mail: f.desmolles@peche63.com; 5 Rovaltain Research Company, 1 avenue de la gare, B.P. 10313, 26958 Valence Cedex 09, France; E-Mail: bcombourieu@rovaltainresearch.com

**Keywords:** blood biomarker, erythrocytes, brown trout, mini-P-gp, P-gp, river pollution

## Abstract

In aquatic organisms, such as fish, blood is continually exposed to aquatic contaminants. Multidrug Resistance (MDR) proteins are ubiquitous detoxification membrane pumps, which recognize various xenobiotics. Moreover, their expression is induced by a large class of drugs and pollutants. We have highlighted the co-expression of a mini P-gp of 75 kDa and a P-gp of 140 kDa in the primary culture of brown trout erythrocytes and in the erythrocytes of wild brown trout collected from three rivers in the Auvergne region of France. *In vitro* experiments showed that benzo[*a*]pyrene, a highly toxic pollutant model, induced the co-expression of mini-P-gp and P-gp in trout erythrocytes in a dose-dependent manner and relay type response. Similarly, in the erythrocytes of wild brown trout collected from rivers contaminated by a mixture of PAH and other multi-residues of pesticides, mini-P-gp and P-gp were able to modulate their expression, according to the nature of the pollutants. The differential and complementary responses of mini-P-gp and P-gp in trout erythrocytes suggest the existence in blood cells of a real protective network against xenobiotics/drugs. This property could be exploited to develop a blood biomarker of river pollution.

## 1. Introduction

The bloodstream is the focal point for the inter-reaction of numerous physiological molecules, essential for life. The bloodstream is the place for accumulation and distribution of xenobiotics, such as drugs and pollutants. Blood cells are continuously exposed to many molecules and must adapt to changing environmental conditions. Thus, any physiological disruption at protein or gene level can be regarded as a “susceptibility” biomarker. Erythrocytes are the major cellular component of blood. In most vertebrates, including mammals and man, mature red blood cells are non-nucleated. However, in birds, reptiles and fishes, mature red blood cells are nucleated [1,2,3]. Fish erythrocytes are exposed to aquatic pollutants [4]. Indeed, pollutants may be found directly in the blood or stored in organs, tissues and cells from which they may be gradually released into the circulation. Changes in the intrinsic parameters of blood cells [5] or in blood biochemistry [6] can be exploited as biomarkers of environmental pollution.

Multidrug Resistance (MDR) proteins are membrane transporters involved in cell defense mechanisms at the cellular level [7]. Indeed, MDR transporters are efflux membrane pumps that transport xenobiotics out of cells. They recognize a large class of substrates with differing structures and properties, such as drugs and pollutants. Therefore, MDR transporters are currently considered as a major element in the mechanism of chemoimmunity [7]. Moreover, MDR proteins are ubiquitous, belonging to the evolutionarily conserved family of the ATP-binding cassette (ABC) proteins found in most living organisms, from bacteria [8], parasites [9,10], free-living protozoa [11,12], aquatic invertebrates and fish [5,13,14,15], *Drosophila* [16,17], plants [18,19] to mammals [7]. In man, the three principal types of MDR proteins identified are: (i) Permeability glycoprotein (P-gp) encoded by *ABCB1* or *MDR1* gene; (ii) MDR-associated protein (MRP) encoded by *ABCC* genes (*ABCC1*, *ABCC2*, plus probably *ABCC3-6* and *ABCC10-11*); (iii) Breast Cancer Resistance Protein (BCRP) encoded by *ABCG2* gene. In man, MDR proteins are essentially known for their role in cancer chemoresistance mechanisms [7]. The target molecules of these efflux pumps are mainly composed of hydrophobic compounds, such as drugs or pollutants. However, they are also able to extrude a wide variety of amphipathic molecules. Indeed, according to many reports in the literature, (i) P-gp preferentially extrudes a wide class of hydrophobic molecules; (ii) BCRP extrudes both hydrophobic drugs and intracellular xenobiotic metabolites and (iii) MRP can extrude hydrophobic drugs, but it is rather involved in the efflux of metabolites like glutathione or glucuronide conjugates [7].

MDR expression (genes/proteins) has been induced in the presence of xenobiotics (drugs and pollutants) [5,7,11,12,16,17]. In man, the response of tumor cells to chemotherapy is often highly variable. Indeed, certain tumors may exhibit intrinsic chemoresistance, which is often associated with intrinsic MDR overexpression in tumor cell membranes. Other tumors may develop chemoresistance during treatment due to increased expression of MDR proteins in response to drugs. This property of MDR transporters may be used as a predictive biomarker. P-gp, which was discovered over thirty years ago, is the most widely studied MDR transporter [20]. It has already been demonstrated that P-gp expression may have prognostic value in several hematological malignancies, including acute myeloblastic and lymphoblastic leukemia [21,22] and multiple myeloma [23]. Moreover, future development in the clinical and therapeutic applications of MDR for cancer treatment, and the challenge of new biomarker development, has been envisaged [24]. In this context, for many years, our research has focused on the development of a biomarker from the expression mode of MDR1/P-gp in organisms responding to environmental pollution, especially by Polycyclic Aromatic Hydrocarbons (PAHs). The latter are ubiquitous environmental contaminants that include highly toxic compounds. These potentially carcinogenic compounds have been found in air, water and food [25,26]. In the freshwater ciliated protozoan *Tertahymena pyriformis*, we have demonstrated the expression of two P-gp of 66 kDa and 96 kDa. The expression of these two P-gp increased in a dose-dependent manner in the presence of increased concentrations of benzo[*a*]pyrene (B*a*P), a genotoxic, cytotoxic and carcinogenic PAH. Similarly, *Tetrahymena P-gp* responded to other PAHs, such as 3-methylcholanthrene, benzanthracene and 7,12-dimethylbenzanthracene (DMBA), and other cancer drugs, such as cyclosporine and adyamincin [11,12]. In *Drosophila melanogaster*, we highlighted the presence of a 140 kDa P-gp encoded by the mdr49-PA gene that was expressed continuously throughout fly development (embryo, larva, pupa and adult) as well as in embryonic *Drosophila* Sl2 cells. In *in vitro* conditions and in the field, *Drosophila* P-gp and mdr49-PA genes were both induced in a dose-dependent response after exposure of flies to air contaminated by various PAHs [16,17]. Furthermore, our recent work has highlighted the presence of a 140-kDa P-gp in brown trout erythrocytes. Moreover, under *in vitro* conditions, P-gp expression in trout erythrocytes increased significantly in a dose-dependent manner after exposure to increased B*a*P concentration [5].

In order to develop a blood biomarker of environmental pollution from MDR proteins, our studies focused on mini-P-gp and P-gp co-expression in the erythrocytes of brown trout and their response to a contaminated medium under *in vitro* conditions and in the field.

## 2. Experimental Section

### 2.1. Fish and Capture

Brown trout (*Salmo trutta fario*) for cell culture were obtained from the “Le Moulin de Pagnat” fish farm in Saint-Saturnin, France.

For field experiments, brown trout (16 to 20 cm) were collected from rivers during the electrofishing campaign carried out by the Departmental Federation for fishing and protection of the aquatic environment of Puy-de-Dôme. Electrofishing was performed according to the AFNOR standard No.T95F N0374.

### 2.2. Anesthesia and Blood Samples

Brown trout were anesthetized in 20 L of river water containing 400 μL of a mixture of clove oil diluted in 2 mL of 70% alcohol. Blood samples were taken by intracardiac puncture into lithium heparinate anticoagulant [5]. Samples were stored at 4 °C at the sampling site and then taken to the lab. Each sample was centrifuged at 800× *g* for 15 min. The plasma and white blood cell fraction were removed and the erythrocyte pellet was washed once with PBS *v:v* and frozen at −80 °C for later protein analysis.

### 2.3. Ethics

In compliance with the European Directive 2010/63/UE and Decree No. 2013-118 dated February 1, 2013 for the protection of animals used for scientific purposes (ref NOR: AGRG12131951D), ethical approval was not required for this study since the fish were anesthetized with an overdose of a natural anesthetic. The authors are affiliated to the Departmental Federation for fishing and protection of the aquatic environment of Puy-de-Dôme and have a license from the “Prefecture of Puy-de-Dôme”—Departmental Directorate of Infrastructure and Agriculture of the Puy-de-Dôme (authorizing the capture and transport of fish for scientific purposes, rescue and/or transfer (regulated by the prefectural order dated December 2009).

### 2.4. Erythrocyte Primary Culture

500 μL of fresh blood were washed twice with 1 mL of PBS with centrifugation at 800× *g* for 10 min. 200 μL of the red blood cell fraction were taken and diluted in 200 μL of Leibovitz’s medium (L-15). From this cell fraction, one million cells per mL were seeded in 25-mL flasks containing 5 mL Leibovitz’s medium (L-15), supplemented with 10% fetal bovine serum, 1% penicillin/streptomycin, 1% fungizone and 1% l-glutamine. The flasks of cell culture were then incubated at a temperature of 21 °C, with stirring at 45 rpm [5].

### 2.5. BaP Solubilization and Cell Culture Exposure

B*a*P was dissolved in dimethyl sulfoxide (DMSO). Then, 10 mL of culture medium with 10^6^ cells/mL were treated with concentrations of 10 μM, 20 μM and 30 μM of B*a*P. The final DMSO concentration remained constant *i.e.*, 0.1%, in all analyzed cell cultures. In parallel, a control culture and a control culture treated with 0.1% DMSO (0.1% DMSO control) were carried out. All experiments were performed at least in triplicate.

### 2.6. Flow Cytometry Analysis of BaP Uptake

B*a*P being a fluorescent molecule, the uptake by red blood cells was measured by FCM using a FACS BD LSRII flow cytometer (Becton Dickinson, Rungis, France) as described in Bamdad *et al.* [12]. For these experiments, red blood cells in primary culture were exposed to 10 μM B*a*P for 1.5 h. PAHs were excited at 355 nm in a LightwaveXcyte at output power of 20 mW. Fluorescence intensities were obtained using a 405 ± 10 nm band-pass filter. For each cytometric parameter investigated, 10,000 events (cells) were analyzed per condition. Mean fluorescence of a given population was expressed in arbitrary fluorescence units (FU) on a log scale. Each experiment was performed at least in triplicate.

### 2.7. MDR Expression Studied by Western Blot Analysis

The Western blot analysis was carried out according to Valton *et al.* [5]. Briefly, the dry pellet, or cell culture pellet, was recovered in 1 mL of lysis buffer (20 mM tris-HCl, 2 mM EDTA, 2 mM EGTA, 6 μM β-mercaptoethanol, and 0.1% protease inhibitor cocktail, pH 7.5). Protein concentrations of samples were determined using the Bradford assay with bovine serum albumin (BSA) as standard. For each analysis, 100 μg·mL^−1^ protein extract were used. For MDR protein detection, the primary monoclonal antibody anti-P-gp C219 (1:500, Calbiochem, San Diego, USA) and for internal control, the mouse monoclonal antitubulin antibody DM1A (1:5000, Sigma, Saint-Quentin Fallavier, France) were used. The secondary antibody was a horseradish-peroxidase (HRP)-coupled goat anti-mouse (1:4000, Promega, Charbonnières-les-Bains, France). The immune complexes were visualized by chemiluminescence (ECL + Western blotting detection reagent, Amersham, Glattbrugg, Suisse) according to the manufacturer’s specifications. The intensities of mini-P-gp, P-gp and control tubulin bands were analyzed by densitometry using Quantity-One software (BioRad, Marnes-la-Coquette, France) by calculating the ratio of the density of each P-gp over the density of the tubulin control. This ratio was expressed in arbitrary units (a.u.).

### 2.8. River Water Chemical Pollutants Analyse

The chemical pollution of three rivers, “Couze Pavin”, “Artière” and “Auzon” in the Auvergne region of France, with gps coordinates N: 45°30'54.7", E: 002°55'24.3"; N: 45°45'10.7", E: 003°07'13.4"; and N: 45°42'53.0", E: 003°11'09.9", respectively, was analyzed. At each collection site, the presence of the following compounds was sought: (i) 17 PAHs (*i.e.*, acenaphthene, anthracene, benzo[*a*]anthracene, benzo[*a*]pyrene, benzo[*b*]fluoranthene, benzo[*ghi*]perylene, benzo[*k*]fluoranthene, chrysene, dibenzo[*a,h*]anthracene, fluoranthene, fluorene, indeno[*1,2,3-cd*]pyrene, 2-methyl-fluoranthene, 2-methyl-naphthalene, naphthalene, phenanthrene and pyrene; (ii) glycophosate and glyphosate and its metabolites aminomethylphosphonic acid; (iii) 348 varieties of pesticides (Supplementary Information, Figure S1).

#### 2.8.1. Extraction Procedure

For PAHs and multi-residue analyses, 10 mg of internal standard triphenylphosphate solution was added to 1 L of river water sample. This solution was then extracted with a mixture of dichloromethane and ethyl acetate (80:20, *v:v*, 3 × 50 mL). The organic phase was evaporated to dryness under a stream of nitrogen and taken up in a mixture of water and acetonitrile (50:50, *v:v*, 1 mL) containing 0.1% formic acid and atrazine-D5 (0.08 mg/L) for internal standard calibration. For glyphosate and AMPA analyses, 2-aminoethyl phosphoric acid was added as internal standard to river water (4 mL). After the formation of fluorenylmethyloxycarbonyl derivatives, the mixture was extracted with diethyl ether for purification.

#### 2.8.2. High Performance Liquid Chromatography (HPLC) Analysis

Liquid chromatography analyses of PAHs were performed on an Agilent 1200 series HPLC system coupled to a fluorescence detector (Agilent Technologies, Waldbronn, Germany). A reversed-phase C18 column was used (4.6 × 250 mM, 5 μM, Waters Corporation, Guyancourt, France). A mixture of water and acetonitrile was used as the mobile phase in gradient elution mode. Excitation and emission wavelengths were chosen specifically for the detection of each PAH.

Multi-residue pesticide analyses were performed on a HPLC system coupled to a high-resolution mass spectrometer (Thermo Ultimate 3000/Bruker QTOF Maxis 4G, Bruker, Marne-La-Vallée, France). Mass spectrometry analyses were performed in positive and negative modes. A reversed-phase C18 column was used (2.1 × 100 mM, 2.2 μM, Thermo). A mixture of water and methanol acidified with 0.01% formic acid and 5% ammonium formate was used as the mobile phase in gradient elution mode.

For glyphosate and AMPA analyses, the aqueous phase was subsequently injected in the HPLC (Agilent 1200 series, Agilent Technologies France, Waldbronn, Germany) with fluorescence detection. The separation was performed on a column NH2 stationary phase (4.6 × 250 mM, 5 μM). A mixture of water and acetonitrile acidified with 0.15% phosphoric acid was used as the mobile phase. Excitation and emission wavelengths were 260 and 310 nM, respectively.

Extraction of internal standard (triphenylphosphate) was quantified for result acceptance. Measurements were all within a 20% variation range of the expected concentration for all analyzed samples.

### 2.9. Statistical Analysis

**Figure 1:**
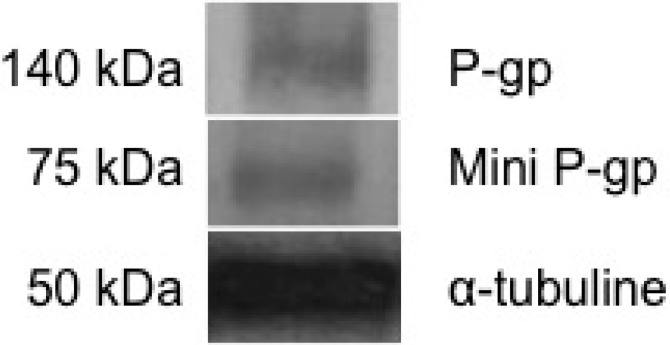
MDR protein expression in brown trout erythrocytes by Western blot analysis. MDR protein expression was studied in total protein extract from dry pellets of brown trout erythrocytes.

Data were expressed as means ± standard deviation of *n* independent experiments. For *in vitro* and field experiments, erythrocytes of five fishes were systematically analyzed. Each experiment was performed at least in triplicate and then statistically compared using a Student’s *t*-test. For *in vitro* experiments, the comparison was applied on each culture condition and on control groups *versus* B*a*P-treated groups. For experiments in the field, mini P-gp and P-gp expression levels were measured in several experiments/specimens and all assays were performed in triplicate. For the flow cytometry experiments, quantitative data were relative *versus* a control, which varies for each experiment. Only one experiment is presented in Figure 1, but we obtained the same pattern three times over. Tests were two-sided and the nominal level of significance was *p* < 0.05 unless otherwise specified.

## 3. Results and Discussion

In aquatic organisms, such as fish, blood is exposed directly to environmental contaminants. Among fish, the brown trout (Salmo trutta fario), a representative species of rivers in France and Europe, provides a ubiquitous and highly sensitive index of water quality. Moreover, MDR proteins are detoxification membrane pumps involved in the efflux of several classes of xenobiotics with different structures and properties, such as drugs and pollutants. Moreover, in contaminated environments, the expression of MDR proteins increases for cell defence against xenobiotics. All these properties make trout erythrocyte MDR proteins an excellent tool for detecting water pollution.

### 3.1. Basal Expression of Two P-gps in Brown Trout Erythrocytes

Basal expression of P-gp was analyzed from a dry pellet of brown trout erythrocytes by Western blotting. The monoclonal anti-tubulin antibody DM1A labelled a tubulin protein with a molecular weight of about 50 kDa. This was used as the internal control (Figure 1).

The monoclonal anti-P-gp antibody C219 clearly recognized two proteins. The first protein having a molecular weight (MW) of 140 kDa was identified as P-gp. The second band presented a MW of 75 kDa and was called mini-P-gp. Expression levels of MDR proteins quantified by Quantity One 1-D Analysis Software (Biorad) were 0.84 ± 0.05 for P-gp and 0.92 ± 0.04 for mini-P-gp. Indeed, the 140-kDa protein has been already identified as P-gp by Valton *et al*. [5], in primary culture of brown trout erythrocytes. However, depending on species, the P-gp molecular weights may be highly variable. Thus, in aquatic organisms, such as other fish, a 165-kDa P-gp has been cited in hepatocyte cell cultures of *Oncorhynchus mykiss* [27] and a fish hepatoma cell line [15]. Moreover, the labeling of an 80 kDa band was previously reported in trout liver and cultured hepatocytes by the C219 monoclonal anti-P-gp antibody [27]. In the freshwater ciliated protozoan *Tetrahymena pyriformis*, two P-gps of 66 kDa and a 96 kDa were identified [11,12]. In the fly model *Drosophila melanogaster*, a P-gp of 140 kDa was cited [16,17]. In mammals, including man, a P-gp of 150 to 170 kDa was identified [7]. Furthermore, the presence of a mini-P-gp of 65 kDA was reported in P388 murine leukemia cells [28]. Similarly, in Natural killer lymphoma, the presence of a short-length P-glycoprotein called mini-P-gp has already been mentioned [29]. To analyze the mode of response of the two P-gps against xenobiotics in trout erythrocytes, the experiments of induction by benzo[*a*]pyrene (B*a*P) were performed under *in vitro* conditions. B*a*P is a genotoxic, cytotoxic and carcinogenic PAH used as a model for studying PAH toxicity [25,26]. It is also a recognized substrate of P-gp in various models [5,11,12,16,17,30,31]. However, it was first necessary to check whether B*a*P penetrated trout erythrocytes.

### 3.2. Benzo[a]Pyrene Uptake by Brown Trout Erythrocytes

**Figure 2:**
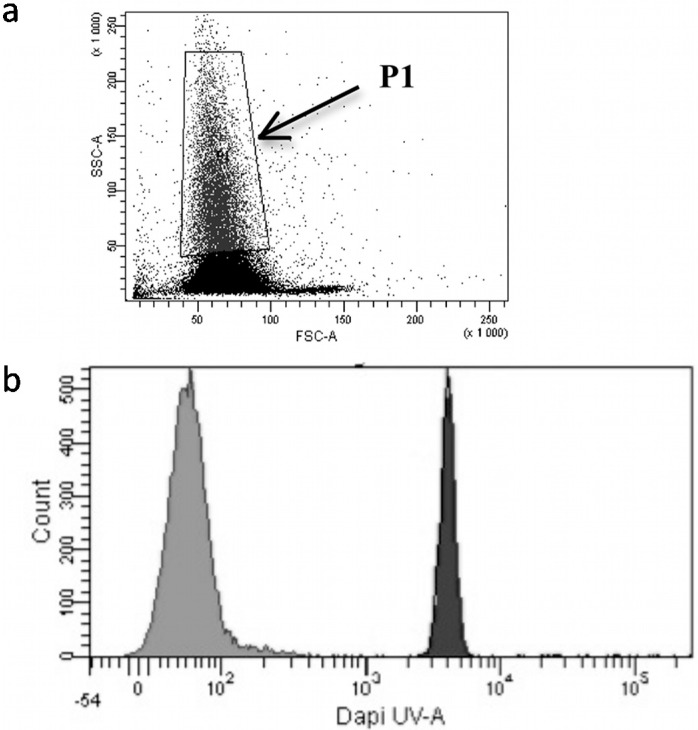
B*a*P uptake in trout erythrocytes analyzed by flow cytometry. (**a**) Flow cytometry (FCM) profile of trout erythrocytes. The gate contained the point-cloud profile of erythrocytes in control cells. Cell size is expressed by forward scatter = FSC-A (*x*-axis), and intracellular structure complexity is expressed by side scatter = SSC-A (*y*-axis). (**b**) Profile of B*a*P-treated trout erythrocytes in primary cultures. B*a*P was naturally fluorescent in UV. Grey histogram: FCM fluorescence profile for control cells and/or 0.1% DMSO control. Black histogram: FCM fluorescence profile of trout erythrocytes treated with 10 μM B*a*P for 1.5 h. FU: Fluorescence Unit (*x*-axis), with cell numbers on the *y*-axis.

B*a*P uptake at 10 μM was followed in trout erythrocyte cell culture for 1.5 h (Figure 2). B*a*P is highly fluorescent and this property was used to follow its uptake into the cells using FCM.

The results (Figure 2) show homogeneous red blood cell profiles between FCMcontrol, 0.1% DMSO control and cells treated with 10 μM B*a*P (Figure 2a). Furthermore, the FCM fluorescence profile (Figure 2b) clearly demonstrated that B*a*P rapidly entered into the red blood cells. In this context, the level of mini-P-gp and P-gp expressed in trout erythrocytes were then analyzed under *in vitro* conditions using the primary culture of trout erythrocytes in the presence of increasing concentrations of B*a*P [5].

### 3.3. Co-Expression of Mini-P-gp and P-gp in Primary Culture of Trout Erythrocytes after Exposure to BaP

**Figure 3:**
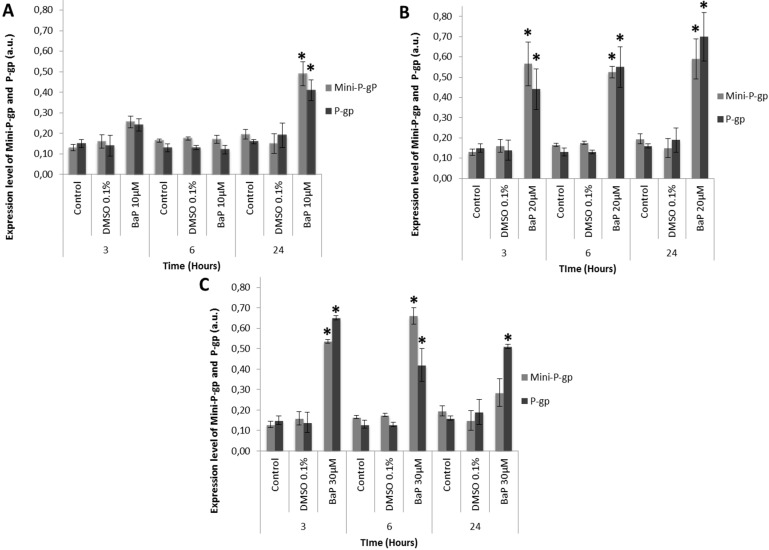
Mini-P-gp and P-gp co-expression in primary cultures of trout erythrocytes in the presence of increasing B*a*P concentrations. Experiments were performed by Western blot analysis. MDR expression level was quantified using Quantity One software (Biorad^®^, publisher, Marnes-la-Coquette, France). a.u.: Arbitrary Units. Red blood cells were treated for 3 h, 6 h and 24 h with (**A**): 10 μM B*a*P; (**B**): 20 μM B*a*P; and (**C**): 30 μM B*a*P. * indicates a significant difference (*p* < 0.05) in mini-P-gp expression and P-gp expression in erythrocyte cultures treated by B*a*P than controls.

The coexpression levels of mini-P-gp and P-gp in trout erythrocytes were then analyzed at 3 h, 6 h and 24 h in the presence of increasing concentrations of B*a*P (10, 20 and 30 μM) using Western blot analysis (Figure 3a–c). Control and DMSO 0.1% control cultures were also performed.

Mini-P-gp expression levels in both control cultures remained relatively constant over time (Figure 3a). The level of mini-P-gp in the control erythrocytes was 0.13 ± 0.02 a.u., 0.17± 0.01 a.u. and 0.20 ± 0.02 a.u. after 3 h, 6 h and 24 h, respectively. The level of mini-P-gp in the 0.1% DMSO control was 0.16 ± 0.03 a.u. for 3 h, 0.18 ± 0.01 a.u. for 6 h and 0.15 ± 0.05 a.u. for 24 h. Similarly, in primary cultures of trout erythrocytes, in both control cells and 0.1% DMSO, P-gp expression levels remained stable during 24 h (0.16 ± 0.01 and 0.19 ± 0.06 a.u., respectively (Figure 3a–c). For mini-P-gp and P-gp, statistical analysis showed no significant difference between control cells and 0.1% DMSO control. Consequently, 0.1% DMSO had no effect the mini-P-gp and P-gp expression in trout erythrocytes.

With 10 μM B*a*P (Figure 3a), the level of P-gp and mini-P-gp in trout erythrocyte remained stable and close to that of controls for the first six hours (0.17 ± 0.03 a.u. for mini-P-gp and 0.12 ± 0.02 a.u. for P-gp). Only after 24 h of B*a*P treatment, mini-P-gp and P-gp expression levels increase significantly with 0.49 ± 0.06 a.u. and 0.41 ± 0.05 a.u., respectively. P-gp induction was about 3-fold higher for mini-P-gp and 2-fold higher for P-gp than for the 0.1% DMSO controls, respectively.

In contrast, in the presence of 20 μM B*a*P (Figure 3a,b), both mini-P-gp and P-gp expression clearly increased throughout the experiment, compared to controls. Indeed, mini-P-gp expression increased clearly and remained high over time, with 0.57 ± 0.11 a.u., 0.53 ± 0.03 a.u. and 0.59 ± 0.11 a.u., after 3 h, 6 h and 24 h, respectively. For the same times, P-gp levels increased also to 0.44 ± 0.1 a.u., 0.55 ± 0.1 a.u. and 0.70 ± 0.1 a.u. respectively. With this B*a*P concentration, mini-P-gp induction presented a mean of about 3-fold higher ratio than control and P-gp expression 2-fold higher ratio than control throughout the experiment.

With 30 μM B*a*P (Figure 3c), mini-P-gp and P-gp expression levels were significantly different in control and treated cultures. A clear induction of the two P-gp expressions was detected in B*a*P-treated cultures. Indeed, mini-P-gp expression levels remained higher during 6 h (0.66 ± 0.04 a.u.) and then fell by half at 24 h (0.29 ± 0.07 a.u.). In contrast, P-gp expression levels remained high during the experiment (0.65 ± 0.01 a.u., 0.42 ± 0.08 a.u. and 0.51 ± 0.01 a.u. after 3 h, 6 h and 24 h, respectively).

All these experiments showed clearly a dose-dependent induction of both mini-P-gp and P-gp in the erythrocytes of brown trout, after exposure to increasing concentrations of B*a*P. However, the responses of mini-P-gp and P-gp seemed to differ in the presence of different concentrations of B*a*P. Indeed, in the presence of a low B*a*P concentration (10 μM), the expression of mini-P-gp and P-gp remained similar and close to controls during the first hours of treatment. Only after 24 h, was a clear induction of the two P-gps detected, when their ratios were three-fold higher than controls (averaging 0.45 ± 0.06 for the two P-gps together). In the presence of higher B*a*P concentrations (20 or 30 μM), both P-gps were clearly induced. At 20 μM B*a*P, mini-P-gp and P-gp were co-expressed similarly over time, at a ratio 3.5-fold higher than controls (averaging 0.56 ± 0.1 for the two P-gps together). At 30 μM B*a*P, mini-P-gp and P-gp were highly co-expressed but with variable levels over time. Indeed, at 3 h, P-gp expression was 1.2-fold higher than mini-P-gp, but this trend was reversed after 6 h, when P-gp expression became 1.5-fold higher. After 24 h, P-gp ratio had again increased to a reach a level 1.8-fold higher than mini-P-gp expression. These results suggest that, in the presence of higher concentrations of B*a*P, the two P-gps play complementary protective roles against the xenobiotic.

In trout erythrocytes, MDR proteins are directly involved in the mechanisms of cellular defense against xenobiotics. For *in vitro* experiments, the B*a*P concentrations (10, 20 and 30 μM) were selected for the following reasons. Firstly, in our previous studies on the B*a*P toxicity in primary culture of erythrocytes, the same B*a*P concentrations were tested and no toxicity was detected, which was probably due to the detoxification action of P-gp [5]. Secondly, similar responses have already been detected in others cell cultures models, *i.e.*, in the ciliated protozoan *Tetrahymena pyriformis* and in SL2 *Drosophila melanogaster* cell line culture in the presence of similar concentrations of B*a*P and other PAHs [11,17,32]. Moreover, in order to compare the co-expression of mini-P-gp and P-gp with our previous results [5], we used the same experimental conditions. Furthermore, for P-gp response against PAHs including B*a*P, similar results have already been described in other models in response to water and air pollution [11,12,16,17]. All these observations in *in vitro* conditions suggest that trout erythrocytes have a cell protection network *via* the expression of these two MDR proteins.

Moreover, according to the literature, P-gp recognizes various molecules with different structures and physicochemical properties, including various drugs and pollutants. Indeed, many PAHs (e.g., B*a*P, 3-methylcholanthrene, benzanthracene, 7.12-dimethylbenzanthracene), heavy metals (e.g., cadmium), pesticides (e.g., diazinon, DDT), insecticides (e.g., chlorpyrifos) and various pharmaceutical drugs (e.g., Vinca alkaloids, actinomycin D, taxol, antracyclines, verapamil) are target substrates of P-gp and can induce its overexpression [7,11,12,16,17,30,31,33,34,35,36,37,38,39]. This property can be exploited for the development of a blood xenobiotic biomarker. To validate this hypothesis, it was necessary to determine whether the two P-gps responded similarly to water pollution in wild brown trout.

### 3.4. Mini-P-gp and P-gp Co-Expression Levels in Brown Trout Erythrocytes from Rivers

**Figure 4:**
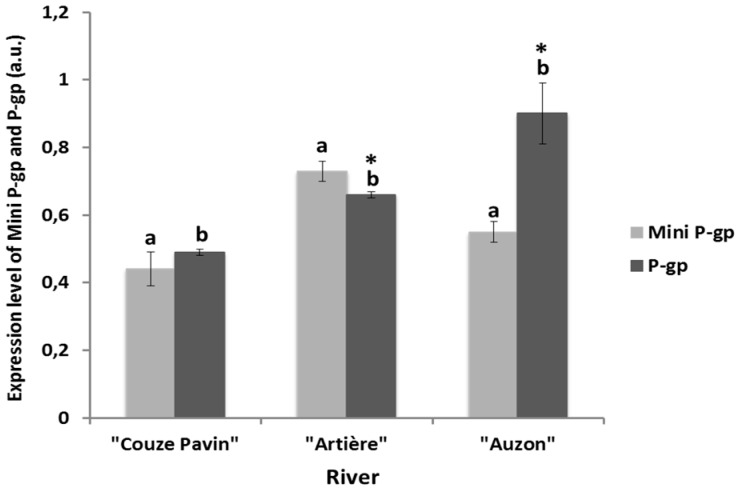
Co-expression of mini-P-gp and P-gp in brown trout erythrocytes from different rivers. The red blood cells were taken from brown trout at sampling sites of different rivers with GPS coordinates: “Couze Pavin” (N 45°30'54.7" E 002°55'24.3"), “Artière” (N 45°45'10.7" E 003°07'13.4") and “Auzon” (N 45°42'53.0" E 003°11'09.9"). Proteins were analyzed in total protein extract from red blood cells using Western blot analysis. P-gp expression was quantified by QuantityOne software (Biorad^®^). a.u.: arbitrary units; a and b indicate a significant difference (*p* < 0.05) of mini-P-gp expression between different rivers and P-gp expression between different rivers, respectively; * indicates a significant difference (*p* < 0.05) between mini-P-gp expression and P-gp expression in each river.

The expression rate of mini-P-gp and P-gp in the erythrocytes of brown trout was then analyzed in three rivers “Couze Pavin”, “Artière” and “Auzon” (Figure 4). These rivers were located in different watersheds in the Auvergne region in France. For these experiments, brown trout blood was collected in the field, conserved at 4°C and Western blot analysis was then carried out in the laboratory. The co-expression levels of the two MDR proteins in the erythrocytes of brown trout collected from the “Couze Pavin” river were 0.44 ± 0.05 a.u. for mini-P-gp and 0.49 ± 0.01 a.u. for P-gp (Figure 4). In the “Artière” river, the MDR co-expression levels were 0.73 ± 0.03 a.u. for mini-P-gp and 0.66 ± 0.01 for P-gp (Figure 4). In the erythrocytes of brown trout from the “Auzon” river, mini-P-gp was 0.55 ± 0.03 a.u. and P-gp was 0.90 ± 0.09 a.u. (Figure 4).

These results showed that mini-P-gp and P-gp were regularly expressed in the erythrocytes of brown trout collected from the three rivers. However, MDR co-expression levels were different for each river. Indeed, the comparison of expression rates of mini-P-gp and P-gp showed that in “Artière” trout, the co-expression levels of both MDR proteins in erythrocytes were about 1.5 times higher than “Couze Pavin” trout. In the brown trout from the “Auzon” river, the P-gp seem to be overexpressed, with a rate 1.8 times higher than in trout erythrocytes from “Couze Pavin” and 1.3 higher than in trout erythrocytes from “Artière”. The differences in the co-expression levels of mini-P-gp and P-gp detected in these rivers may be related to the degree of water pollution. Therefore, the type of contamination of the river water was then investigated. For this, we chose to check for the presence of PAHs, glyphosate and AminoMethylPhosphonic Acid [40] and multi-residue pesticides.

### 3.5. Pollution Analysis of the River Water

The degree of river water pollution was then analyzed at the same collecting sites (Table 1).

**Table 1:** Organic micropollutants analyses in different rivers located in different watersheds. The level of water pollution of the “Couze Pavin”, “Artière” and “Auzon” rivers was analyzed in parallel at the same trout collecting sites. PAH: Polycyclic Aromatic Hydrocarbon, AMPA: Aminomethylphosphonic Acid, DCPMU: 1-(3,4-DiCloroPhenyl)-3-Methyl Urea, LOQ: limit of quantification (concentration for which the compound signal is at least 10 times the background signal intensity).

River	GPS Coordinates of Sampling Sites	PAH (μg/L)	Multi-Residue Pesticide (μg/L)						
		Acenaphtene	AMPA	Oxadiazon	Atrazine	Coumatetralyl	Diuron	DCPMU	Myclobutanil
Couze Pavin	N 45°30'54.7" E 002°55'24.3"	0.030	-	-	-	0.009	-	-	-
Artière	N 45°45'10.7" E 003°07'13.4"	-	0.120	0.021	<LOQ	-	0.005	0.008	-
Auzon	N 45°42'53.0" E 003°11'09.9"	-	0.100	0.017	<LOQ	0.022	<LOQ	<LOQ	0.005

At each sampling site, the presence of: (i) 17 different PAHs, glycophosate and its metabolite AMPA and (ii) 348 varieties of pesticide were sought (Supplementary Information, Figure S1).

In “Couze Pavin” river (N: 45°30'54.7"; E: 002°55'24.3"), only one PAH, Acenaphthene at 0.030 μM and one multi-residue pesticide, Coumatetratyl at 0.009 μg/L, were detected.

In “Artière” river, (N: 45°45'10.7"; E: 003°07'13.4"), the presence of several multi-residue pesticides, such as AMPA at 0.120 μM, Oxadiazon at 0.021 μM, Diuron at 0.05 μM and its metabolite 1-(3,4-DiCloroPhenyl)-3-Methyl Urea (DCPMU) at 0.08 μM, plus traces of Atrazine Quantification Limit (QL), were found.

In “Auzon” river, (N: 45°42'53.0"; E: 003°11'09.9"), the presence of several multi-residue pesticides, such as AMPA at 0.1 μM, Oxadiazon at 0.017 μM, Coumatetratyl at 0.022 μM and Myclobutanil 0.005 μM, plus traces of Atrazine, Diuron and DCPMU (QL), were detected.

There was a clear difference in the degree of river contamination. “Couze Pavin” seemed to be less contaminated than “Artière” and “Auzon”. Secondly, the nature of the pollutants differed in each river. Similarly the co-expression levels of mini-P-gp and P-gp were different and seemed to be correlated to the type of water contamination. Indeed, in “Couze Pavin” that was contaminated by one PAH, Acenaphthene, and an anti-vitamin K anticoagulant rodenticide [41] Coumatetratyl, the erythrocytes expressed similar levels of mini-P-gp and P-gp (mean 0.45 ± 0.07 a.u. and not significantly different). In the erythrocytes collected from brown trout in the “Artière” river which was contaminated with several herbicides, such as AMPA, Oxadiazon, Diuron, DPCMU and Atrazine (traces), the expression levels of mini-P-gp (0.73 ± 0.03) and P-gp (0.66 ± 0.01) were significantly different, but these levels were 1.7 and 1.3 times higher than mini- P-gp and P-gp in the erythrocytes of brown trout from “Couze Pavin”. These results suggest that the both MDR proteins may reflect the degree of river pollution. The “Auzon” river was contaminated by some pollutants common to the other two rivers (Oxadiazon, Coumatetratyl, Atrazine, Diuron and DCPMU) plus the fungicide Myclobutanil. Nevertheless, the overall pollution of “Auzon” river was higher than both “Artière” and “Couze Pavin” rivers. While mini-P-gp expression remained similar to that of the other rivers, the P-gp expression level clearly increased with a level 1.3 times higher than “Artière” and 1.8 times higher than “Couze Pavin”. These results suggest that an increased river pollution level can lead to increased expression of P-gp. Furthermore, previous studies have clearly reported that other anticoagulants than Coumatetratyl, such as Vitamin K antagonists, are substrates of P-gp [5,42,43].

The principal physiological function of MDR proteins is to provide general protection against xenobiotics at the cellular level [11,12,13,16,17,34,41,42,43,44,45]. Our results obtained (i) *in vitro* using primary trout erythrocytes culture and (ii) in erythrocytes from river fishes, suggest that mini-P-gp and P-gp are both involved in cellular defence against xenobiotics with complementary protective roles. Indeed, *in vitro* experiments in trout erythrocytes clearly showed that B*a*P, a toxic PAH pollutant, induced increasing expression levels of mini-P-gp and P-gp. Moreover, during B*a*P exposure, the expression level of these two MDR proteins appeared to be increased in turn over time, depending on the PAH concentration. Indeed, mini-P-gp seems to be overexpressed first, and then P-gp takes over mini-P-gp. In river trouts, expression levels of the mini-P-gp and P-gp may reflect the degree of contamination of the river at the time of blood sampling. The overall pollution degree of “Auzon” is higher than “Artière” and the latter exceeds that of “Couze Pavin”. In parallel, in “Auzon” P-gp was expressed at a higher level than mini-P-gp in erythrocytes. In “Artière”, which is less polluted, P-gp was expressed at a lower level than mini-P-gp in erythrocytes. Finally, in “Couze Pavin”, with very low pollution, a similar expression was detected for both MDR proteins. Once again, these results confirmed the relay role of mini-P-gp and P-gp in wild brown trout erythrocytes, which was also observed in *in vitro* conditions. Depending on both the overall degree of river pollution and the type of contaminants, erythrocytes have the ability to modulate the expression of both MDR proteins. The complementary responses of mini-P-gp and P-gp in trout erythrocytes suggest the existence of a real protective network against xenobiotics/drugs in blood cells.

## 4. Conclusions

Brown trout erythrocytes co-express two MDR proteins, mini-P-gp and P-gp, which may be involved in the defence mechanism of red blood cells against xenobiotics. *In vitro* studies using primary culture of erythrocytes and erythrocytes collected from wild trout from three different rivers highlighted the possible protective response of both MDR proteins involving a relay mechanism. Indeed, according to the overall degree of overall river pollution and to the nature of the pollutants, mini-P-gp and/or P-gp expression was increased, probably as part of a blood cell defense mechanism. All these results suggest that co-expression of mini-P-gp and P-gp in trout erythrocytes could be exploited as an early blood biomarker for detecting the overall degree of river pollution. Indeed, the principle of MDR response is based on a defense mechanism at the cellular level against xenobiotics, measured in living organisms. Therefore, this evaluation could serve as an indicator to prevent acute contamination. Moreover, the MDR proteins respond to many different contaminants. This property could be used as a tool for timely monitoring of overall water pollution before physico-chemical analyzes.

## Supplementary Files

Supplementary File 1
