# Supplementary figures and images for: Mini-P-gp and P-gp Co-Expression in Brown Trout Erythrocytes: A Prospective Blood Biomarker of Aquatic Pollution
Source: Diagnostics (Basel). 2015 Jan 12;5(1):10–26. doi: 10.3390/diagnostics5010010 (PMC4665547; doi:10.3390/diagnostics5010010)

Supplementary Information

**Figure S1.** List of 348 pesticides analyzed in multi-residue analysis.


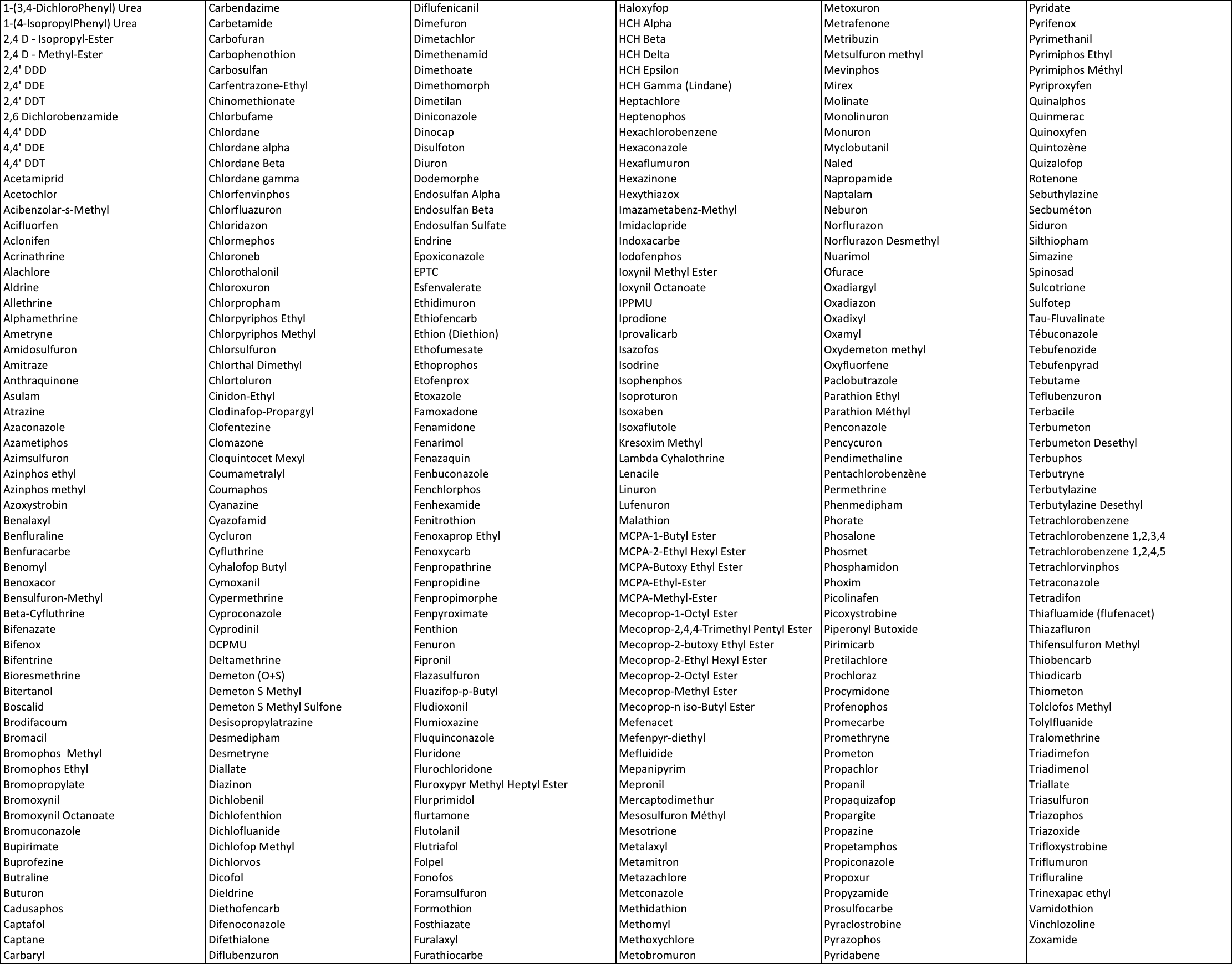

Supplement: Supplementary File 1 [file diagnostics-05-00010-s001.docx]
